# Abdo-Man: a 3D-printed anthropomorphic phantom for validating quantitative SIRT

**DOI:** 10.1186/s40658-016-0151-6

**Published:** 2016-08-05

**Authors:** Jonathan I. Gear, Craig Cummings, Allison J. Craig, Antigoni Divoli, Clive D. C. Long, Michael Tapner, Glenn D. Flux

**Affiliations:** 1 Joint Department of Physics, The Royal Marsden NHS Foundation Trust and Institute of Cancer Research, Sutton, Surrey UK; 2 Research and Development, Sirtex, North Sydney, Australia

**Keywords:** SIRT, 3D printing, Phantoms, Quantification, Dosimetry, Microspheres

## Abstract

**Background:**

The use of selective internal radiation therapy (SIRT) is rapidly increasing, and the need for quantification and dosimetry is becoming more widespread to facilitate treatment planning and verification. The aim of this project was to develop an anthropomorphic phantom that can be used as a validation tool for post-SIRT imaging and its application to dosimetry.

**Method:**

The phantom design was based on anatomical data obtained from a T1-weighted volume-interpolated breath-hold examination (VIBE) on a Siemens Aera 1.5 T MRI scanner. The liver, lungs and abdominal trunk were segmented using the Hermes image processing workstation. Organ volumes were then uploaded to the Delft Visualization and Image processing Development Environment for smoothing and surface rendering. Triangular meshes defining the iso-surfaces were saved as stereo lithography (STL) files and imported into the Autodesk® Meshmixer software. Organ volumes were subtracted from the abdomen and a removable base designed to allow access to the liver cavity. Connection points for placing lesion inserts and filling holes were also included.

The phantom was manufactured using a Stratasys Connex3 PolyJet 3D printer. The printer uses stereolithography technology combined with ink jet printing. Print material is a solid acrylic plastic, with similar properties to polymethylmethacrylate (PMMA).

**Results:**

Measured Hounsfield units and calculated attenuation coefficients of the material were shown to also be similar to PMMA. Total print time for the phantom was approximately 5 days. Initial scans of the phantom have been performed with Y-90 bremsstrahlung SPECT/CT, Y-90 PET/CT and Tc-99m SPECT/CT. The CT component of these images compared well with the original anatomical reference, and measurements of volume agreed to within 9 %. Quantitative analysis of the phantom was performed using all three imaging techniques. Lesion and normal liver absorbed doses were calculated from the quantitative images in three dimensions using the local deposition method.

**Conclusions:**

3D printing is a flexible and cost-efficient technology for manufacture of anthropomorphic phantom. Application of such phantoms will enable quantitative imaging and dosimetry methodologies to be evaluated, which with optimisation could help improve outcome for patients.

## Background

Selective internal radiation therapy (SIRT) with Y-90 microspheres is a radiotherapy option for the treatment of liver tumours from both primary liver cancer (HCC) and liver metastases arising from various primaries including colorectal and breast cancer. Liver tumours are fed primarily with blood flow from the hepatic artery while normal liver parenchyma is fed primarily from the portal vein [1]. To exploit this property, Y-90 microspheres are administered by injection through a trans-femoral catheter positioned in the hepatic artery. The microspheres, which are sized so as to lodge in the neovascular rim of the lesion, are then selectively concentrated in the tumour following administration.

The use of SIRT is rapidly increasing, and the need for quantification and dosimetry is becoming more widespread to facilitate treatment planning and verification [2]. Following administration of the microspheres, a SPECT-CT or PET-CT scan is performed to assess the Y-90 distribution. Y-90 bremsstrahlung SPECT-CT scanning provides low image quality and poor quantitative accuracy [3]. PET-CT can be used for Y-90 imaging with improved resolution [4] and higher accuracy for quantification [5] than SPECT-CT. However, the branching ratio for pair production is very low at only 3.2 × 10^−5^ resulting in long scan times and low count data. Image analysis is generally performed using relatively simple geometrical phantoms which are designed to evaluate given imaging phenomena, characteristics or correction methods. Anatomical phantoms are useful for providing more general qualitative and quantitative estimates of clinical image quality or for analysis of complex image processing regimens (such as a dosimetry protocol) [6–10]. However, anatomical phantoms are generally more expensive, and current commercial phantoms do not adequately represent the microsphere uptake distributions observed in SIRT patients. To better understand the merits of imaging methodologies for Y-90 SIRT and the application of quantitative imaging for dosimetry, a phantom that represents the patient cohort would be greatly beneficial.

Recent work using rapid prototyping has demonstrated that 3D printing offers flexibility in design at a reduced cost in comparison with traditional phantoms [10]. Commercially available printers are generally based on three main techniques: thermoplastic extrusion, powder deposition and stereolithography. Thermoplastic deposition uses a heated nozzle to extrude small beads of thermoplastic material. As the material hardens, new layers are built up to create a 3D object. This method is employed in low-cost printers but lacks the resolution and flexibility of some of the other techniques. Powder deposition printers apply thin layers of binding material on the printer tray and then coat this with a thin layer of powder. This process is repeated to build up the powder/binder layers to create a 3D object. This technique generally offers higher resolution than the extrusion technique. However, the final material is brittle and porous, requiring additional sealing for long-term use. Stereolithography-based printers employ a vat of light-curable resin and a laser light to build parts. The laser beam traces a cross section of the object on the surface of the liquid resin. Exposure to the laser light solidifies the pattern and joins it to the layer below, the resolution achievable is of the order of a few microns and the final build material is more durable than other 3D printing techniques. These printers are now used for final production parts and can produce fine-resolution structures on a sufficient scale to create bespoke molecular imaging test objects.

In this study, we describe the design and manufacture of a bespoke phantom (Abdo-Man) for quantitative imaging analysis of SIRT. A patient-realistic torso phantom was developed with liver and lung organs and multi-positional lesions. The phantom is based on anatomical information obtained directly from MRI data and printed using a Stratasys Connex3 3D printer.

## Methods

### Phantom design

Key criteria in the design of the imaging phantom were considered. The phantom should be anatomically realistic and simulate a patient abdomen, both visually and when imaged with scintigraphy and x-ray computed tomography. A fillable section within the structure was required to represent activity distributions within a liver. The liver section needed to accommodate multiple inserts for lesion representation and allow flexibility in insert arrangement while allowing reproducible assembly for repeated studies. All materials used in the phantom must have similar densities and attenuation coefficients to tissue. The material for the lesions should also be transparent for visualisation and ease of filling. Filling and assembly should be uncomplicated to reduce radiation exposure when preparing the phantom. When filled, the material should have low water absorption, be water tight at all seals and be sufficiently strong to maintain structural integrity when filled and transported. Finally, a total weight limit to the phantom was specified as 20 kg to ensure transportation and manual handing constraints were met.

**Fig. 1:**
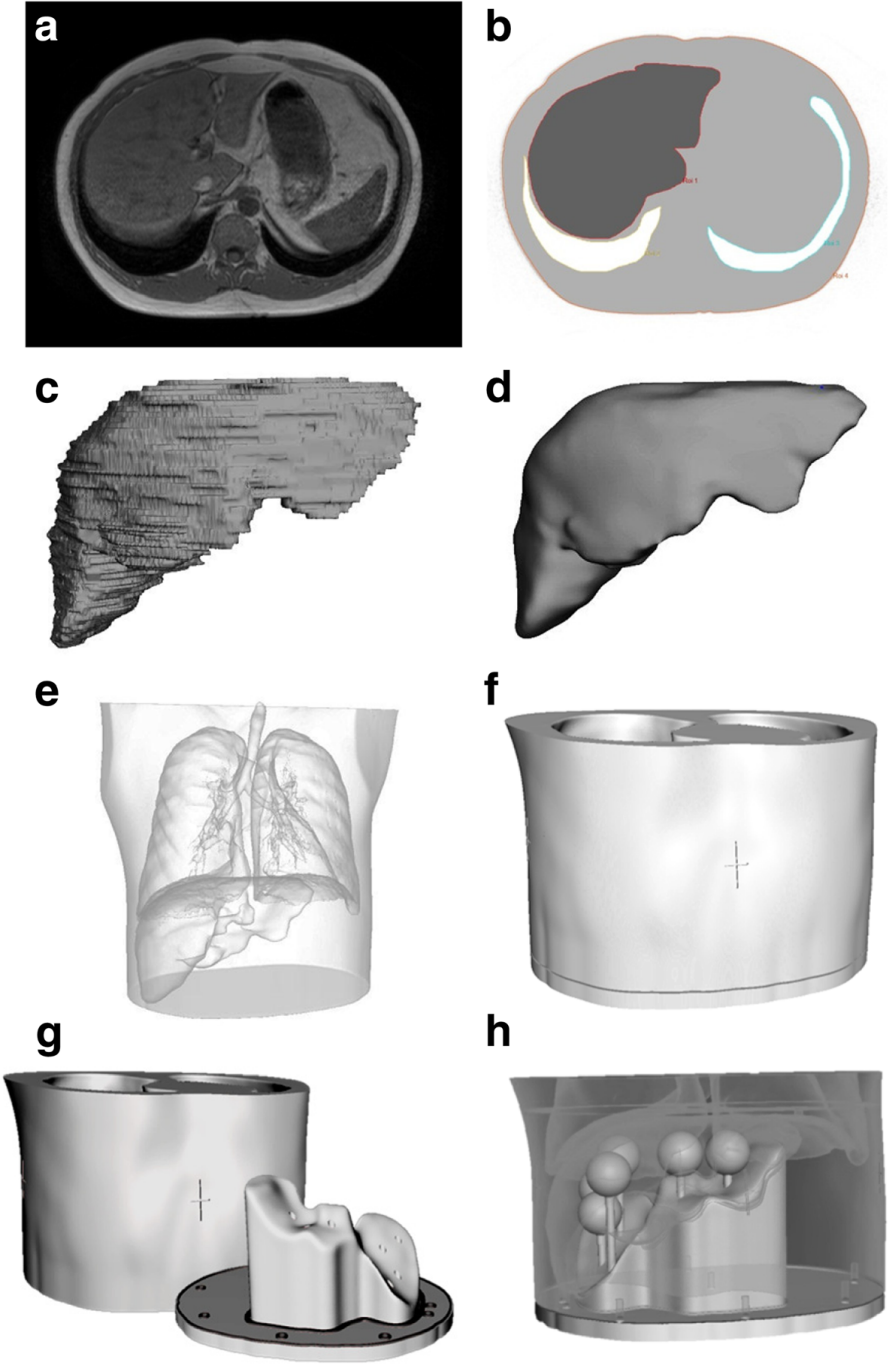
Transaxial slice of the MR scan (**a**) and segmented organs (**b**). 3D visualisation of the liver (**c**) and smoothed liver (**d**). Transparent view of the torso with liver and lung anatomy (**e**). Completed phantom design assembled (**f**) and with base removed (**g**). Transparent view of the phantom showing base and lesion inserts (**h**)

**Fig. 2:**
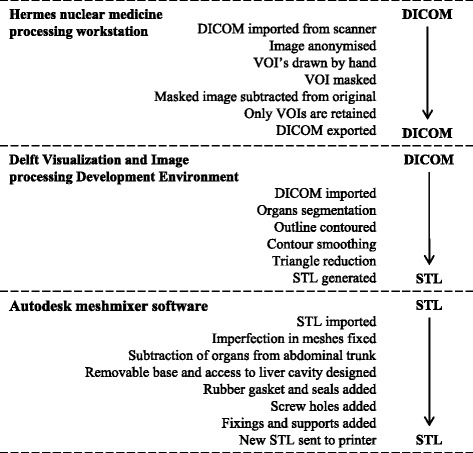
Flow chart of the image processing methodology

Mean liver volume of patients undergoing SIRT were taken from that measured by Theysohn et al. [11]. The abdomen of a 32-year-old male volunteer with an appropriate liver volume and anatomy for representation of the patient cohort was then selected. Anatomical data were obtained from a 24-s T1-weighted volume-interpolated breath-hold examination (VIBE) on a Siemens Aera 1.5 T MRI scanner, giving an in-plane pixel size of 0.7- and 2.8-mm contiguous slices. The required organ volumes were generated from the anatomical dataset and converted to the appropriate file format using a methodology similar to that previously described [10]. Organs were delineated and segmented on the Hermes Hybrid Viewer 2.2c image processing software (Stockholm, Sweden) to create a new dataset containing only the required outlined volumes (liver, lungs and abdominal trunk). Figure 1a, b shows the original MR slice and segmented organ outlines. Organ volumes were exported to the Delft Visualization and Image processing Development Environment (DeVide) [12] for smoothing and surface rendering (Fig. 1c). To remove the MR pixelation, the 3D surface mesh was smoothed (Fig. 1d) and saved as a binary stereo lithography (STL) file. The STL files were imported into the Autodesk Meshmixer software (Autodesk Inc.) and the organ volumes subtracted from the abdominal trunk, to create a fillable liver cavity. To ensure sufficient wall thickness in the phantom between the liver and the lungs, the liver volume was relocated 5 mm in an inferior direction prior to subtraction from the main body. A removable base was designed to allow access into the liver cavity and connection points positioned for placing lesion inserts. A flow diagram illustrating the image processing procedure is given in Fig. 2, indicating the software tools used and data file type at each processing step. Unlike previous designs [10], which use a modular assembly of fillable organ shells, the solid abdominal trunk with liver void of the Abdo-Man phantom means the phantom is more robust and should be less prone to damage during transport and filling.

**Fig. 3:**
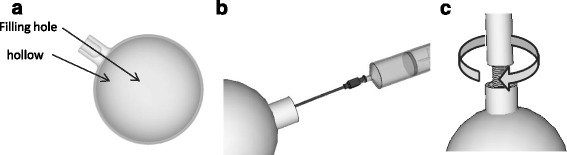
Schematic illustration indicating lesion design (**a**), lesion filling (**b**) and connection port (**c**)

**Fig. 4:**
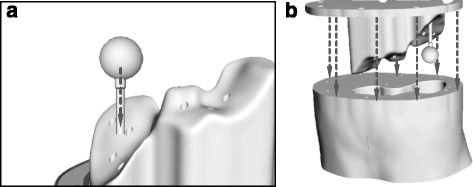
Schematic illustration indicating how lesion and support rods are placed within the phantom base (**a**) and how the base is fitting into the main phantom body (**b**)

Spherical lesion inserts were designed for insertion into the finished phantom using the Meshmixer software. Spheres with diameters of 10, 20, 30, 40 and 50 mm were designed with 1-mm wall thicknesses. Spheres were designed to be connected to the base with detachable support rods which attach to the spheres via connection ports with M6 screw fittings. One-millimetre holes at the connection points on the spheres allow the inserts to be emptied or filled with a 4-in. (102 mm) 19-gauge needle, and the hole is then sealed when the support rod is connected. Figures 3 and 4 illustrate the sphere designs and how they are assembled within the phantom. Once assembled, the liver void can be filled via an access port in the base of the phantom. For consecutive acquisitions with varying concentrations in the liver, addition activity can be added as necessary. This procedure is quicker and simpler than required by alternative designs whereby the phantom may need to be dismantled to access the liver section.

In addition to simple spheres, more complex inserts were also designed, including:

- Forty-millimetre hollow sphere with 25-mm solid inner sphere to represent the deposition of microspheres in the neovascular rim of the tumour around a necrotic core.
- Forty-millimetre hollow sphere with the outer rim being divided into two compartments. This represents lesions where arterial feeding happens through different arterial networks—such as the left hepatic and right hepatic arteries.
- Forty-millimetre internal sphere where the external shell has a 1-cm circular area which is entirely blocked off. This simulates small regions of a lesion where microspheres are not deposited.
- Forty-millimetre-diameter hollow sphere, 1-mm wall thickness, with internal hollow sphere also with 1-mm wall thickness and internal diameter of 25 mm. Each sphere can be filled independently.

**Fig. 5:**
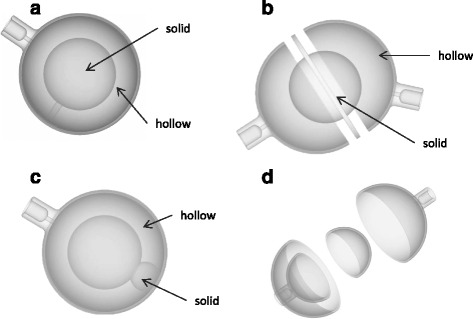
Schematic illustrations of lesion designs, to represent the deposition in the neovascular rim (**a**), feeding through different arterial networks (**b**), region of tumour where microspheres are not deposited (**c**) and inhomogeneous deposition at different concentrations (**d**)

Schematic images of these inserts are shown in Fig. 5.

### Phantom production

The phantom was printed using a Connex3 PolyJet printer (Stratasys Ltd., Eden Prairie, MN, USA). A 16-μm layer of liquid ultraviolet-curable photopolymer is printed onto the build tray. An ultraviolet laser then cures the resin solidifying the pattern traced on the tray. This process is then repeated for each layer. Where overhangs or domed shapes are required, a removable support material is printed on the under layers to prevent the structure collapsing before curing. Various photopolymer resins are available for printing; in this case, a white opaque resin was chosen for the main phantom body (VeroWhite Plus FullCure 835). A black rubber-like material (TangoBlack Plus FullCure 980 Shore 27a) was printed alongside the main phantom material to create gaskets to seal the phantom around the base and screw fittings. Lesion inserts were printed using a transparent polymer, (VeroClear FullCure 810) to enable liquid level to be observed during filling.

### Material properties

To test the suitability of the photopolymers prior to printing, material properties reported by the manufacturer were compared to those more commonly used in phantom production. In addition, cubic test objects were printed and the density and CT Hounsfield units measured. Composition of the print material has previously been reported as a mixture of acrylic monomers and oligomers, with a small proportion (<2.5 %) of a photo-initiator [10]. As the photo-initiator is subject to intellectual property, no information regarding elemental composition is available. An estimate of material attenuation at isotope energies was estimated assuming that the monomer/oligomer mixture has a similar effective atomic number to polymethylmethacrylate (PMMA) and substituting the unknown initiator for materials with different effective atomic numbers as an input into the NIST X-COM program [13]. The effective atomic number of the unknown initiator was increased until the outputted material attenuation corresponded to that measured on CT.

### Phantom geometry

To verify that the phantom was a true representation of the original anatomy, comparisons were made against the original MR dataset. Post production, the volume of water required to fill the phantom was compared to the outlined volume measured on MR. X-ray CT images of the phantom were also acquired and a visual inspection of the CT and MR datasets performed. Transaxial slices through the liver section were compared and diametrical measurements of the liver and abdominal trunk made using the Hermes Hybrid Viewer 2.2c image processing software (Stockholm, Sweden).

### Phantom imaging and dosimetry

To demonstrate the application of the phantom, multimodality imaging was performed with Y-90 SPECT/CT bremsstrahlung, Y-90 PET/CT and Tc-99m SPECT/CT. Three different lesion designs were used within the phantom: a 20-mm sphere, a 40-mm sphere and a 40-mm hollow sphere with 25-mm solid inner sphere. For the Y-90 imaging, the liver section of the phantom was filled with 500 MBq of Y-90 chloride, mixed with 0.2 g of disodium ethylenediaminetetraacetic acid (EDTA) injection to ensure a uniform mixture at 0.29 MBq/ml. Lesion inserts were filled with the appropriate concentration of Y-90 solution (1.72 MBq/ml) to give a final liver-to-lesion concentration ratio of 1:6. Y-90 activities were determined from a stock solution measured under calibration conditions with a Fidelis secondary standard dose calibrator. Dilution activities and subsequent concentrations were determined using accurate mass measurements made during dispensing. A similar procedure was carried out to prepare the phantom for Tc-99m imaging using a total activity of 200-MBq Tc-99m pertechnetate.

Y-90 PET/CT imaging of the phantom was performed as described by Willowson et al. [14] on a Siemens Biograph mCT scanner using a Na-22 isotope selection (as Y-90 was not an available option). Two bed positions acquired at 15 min were sufficient to cover the phantom length. Images were reconstructed using an ordered subset expectation maximization (OSEM) iterative reconstruction algorithm, 2 iterations and 16 subsets, with TOF and PSF correction. The final image size was a 200 × 200 matrix with 4-mm voxels smoothed with a 4-mm Gaussian kernel.

Y-90 bremsstrahlung imaging was performed on a Siemens Symbia Intevo SPECT/CT scanner fitted with medium-energy general purpose collimators. Acquisitions were acquired with 72 projections at 20 s each. Energy window settings were chosen based on the work by Heard et al. [15] and covered an energy range of 56–268 keV. Images were reconstructed with an OSEM iterative reconstruction algorithm, 4 iterations and 8 subsets, with a PSF correction and CT attenuation correction. Ideally, PSF correction would be based on a measured bremsstrahlung PSF; however, this was not available in this version of reconstruction software. Instead, a theoretical 2D Gaussian kernel, adjusted for septal penetration, is applied based on the centroid energy of the window and the medium-energy collimator.

Tc-99m SPECT/CT of the phantom was carried out to demonstrate the comparative image quality of MAA over therapy imaging. SPECT/CT was performed using a similar protocol to the bremsstrahlung imaging using LEHR collimators and a 15 % energy window centred at 140 keV.

Image analysis and absorbed dose calculations for all three lesions and imaging methodologies were performed using the partition model [16] and in three dimensions using the local deposition method [17]. For the bremsstrahlung and Tc-99m imaging, quantification was achieved using the total counts within the liver and the known phantom activity. Quantification of the PET imaging was performed using the inbuilt calibration factors and scaling the reconstructed image according to the known branching ratio of Y-90 and Na-22. Measured absorbed dose distributions were compared to a “reference dose distribution” derived from the known activity in each phantom compartment and the OEDIPE [18] dosimetry interface tool for MCNPX2.5 Monte Carlo (MC) simulations. Throughout all filling and scanning protocols, finger and body TLDs were worn as standard practice. No excess doses to the operators were reported by the radiation dosimetry service.

## Results

### Phantom design

**Fig. 6:**
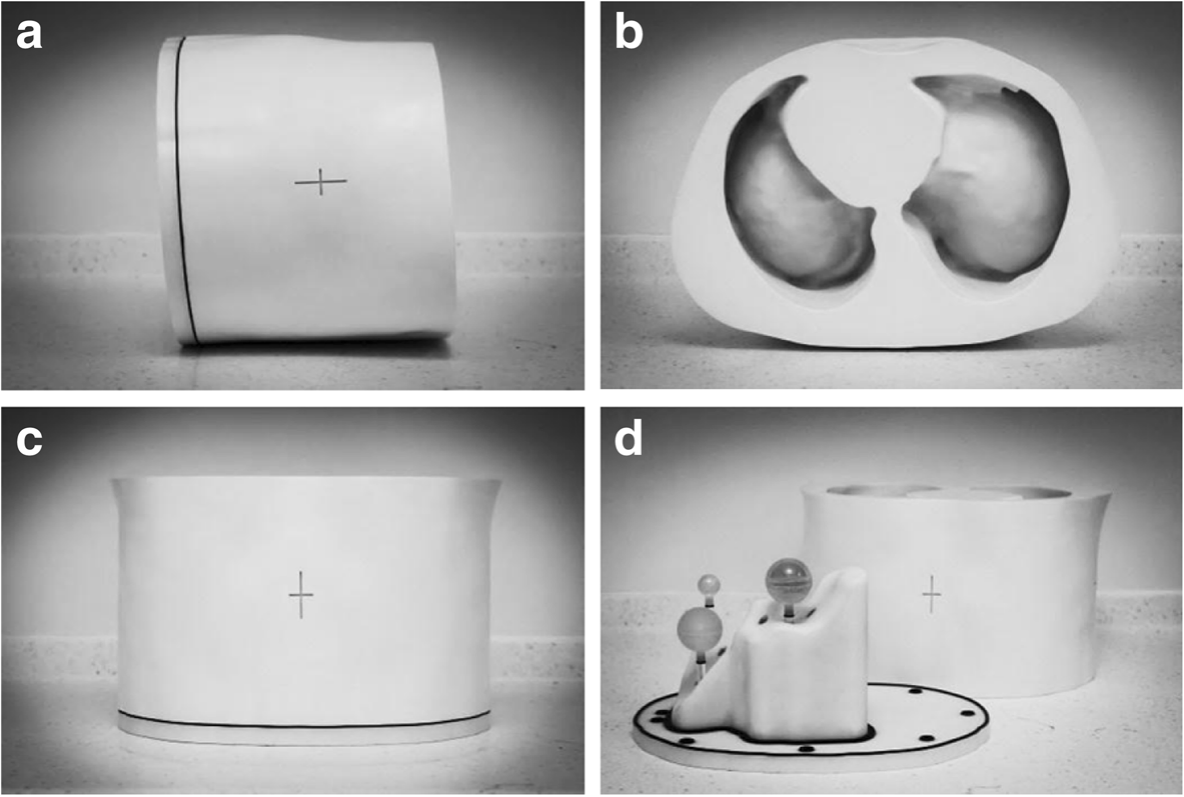
Photographs of the constructed phantom (**a**–**c**). Photograph of the phantom with the base and lesion inserts separated from the main torso (**d**)

A photograph of the completed phantom is shown in Fig. 6a–c. The removable base with an example arrangement of lesion inserts is shown in Fig. 6d.

### Phantom production

Material consumption and print time for the main phantom body, base and lesion inserts are summarised in Table 1. The total print time of any 3D object is dependent on size, and larger objects took several days to print although as the printing process is automated, this required no intervention. The only restriction on this is the print cartridge size (3.6 kg) which required multiple changes for printing of the larger objects. Material cost varies from €0.10 to €0.30 excluding VAT depending on the material being printed. Total production cost for the project was less than €11,000 which compares favourably with costs of commercially available anthropomorphic phantoms. Costs of printers vary from €10K to €250K depending on printer size and material compatibility. Bureau services are available for outsourcing printing projects.

**Table 1:** Material consumption and print time for different organs and lesion inserts

Organ	Material	Consumption (g)	Print time (h)
Main body	Opaque (white)	16,309	109
	Rubber-like	139	
	Support	2572	
Base	Opaque (white)	3480	38
	Rubber-like	95	
	Support	424	
Lesions (total)	Transparent	244	7
	Support	433	

### Material properties

Measured Hounsfield units (HU), densities and estimated linear attenuation coefficients for common isotope energies are summarised in Table 2. No significant difference between the PMMA, transparent material and opaque material was observed on CT, indicating an equivalent attenuation at the CT energy range (*μ* = 0.21 cm^−1^ at 60 keV). Material density was measured at 1.18 g/cm^3^ for the solid materials and 0.9 g/cm^3^ for the rubber-like material. The lower density of the rubber material explains the lower HU of 96 measured for this material.

**Table 2:** X-ray properties of different materials

			Attenuation coefficient (cm^−1^)				
Material	Density (g/cm^3^)	Measured HU	140 keV (Tc-99m)	171 keV (In-111)	254 keV (In-111)	365 keV (I-131)	511 keV (PET)
Water	1.00	0	0.15	0.14	0.13	0.11	0.096
Transparent	1.18	127 ± 15	0.17	0.16	0.15	0.13	0.110
Opaque (white)	1.18	127 ± 15	0.17	0.16	0.15	0.13	0.110
Rubber	0.90	96 ± 15	0.13	0.14	0.15	0.12	0.084
PMMA	1.18	126 ± 15	0.17	0.16	0.15	0.13	0.110

Physical properties of the print materials reported by the manufacturer are summarised in Tables 3 and 4 with comparison to other commonly used materials in phantom manufacture. The print material was found to be less brittle than PMMA and therefore less prone to shattering or breaking under strain. Water absorption is reportedly higher than PMMA, so that it is more prone to swelling when submerged. However, this is still comparable to other plastic materials, such as nylon with water absorption of up to 8.5 %. No discernible swelling or functional deformation in the material was observed after submergence for 72 h.

**Table 3:** Physical properties of the print material and other common plastics

Material	Tensile strength (MPa)	Elongation at break (%)	Modulus of elasticity (MPa)	Flexural strength (MPa)	Water absorption (%)	Rockwell hardness	Density (g/cm^3^)
Transparent	50–65	10–25	2000–3000	75–110	1.1–1.5	M73–M76	1.17–1.18
Opaque (white)	50–65	10–25	2000–3000	75–110	1.1–1.5	M73–M76	1.18–1.19
PMMA	55–76	2	2400–3400	82–117	0.3	M80–M100	1.18
Nylon 6/6	85	90	2800	117	8.5	M88	1.14

**Table 4:** Physical properties of the rubber print material and nitrile rubber

Material	Tensile strength (MPa)	Elongation at break (%)	Compressive set (%)	Shore hardness	Tensile tear resistance (kg/cm)	Density (g/cm^3^)
Rubber-like	0.8–1.5	170–220	4–5	A26–A28	2–4	1.12–1.13
Nitrile Buna rubber	1.4–17	350–650	30	A65	4	1.20

### Phantom geometry

**Fig. 7:**
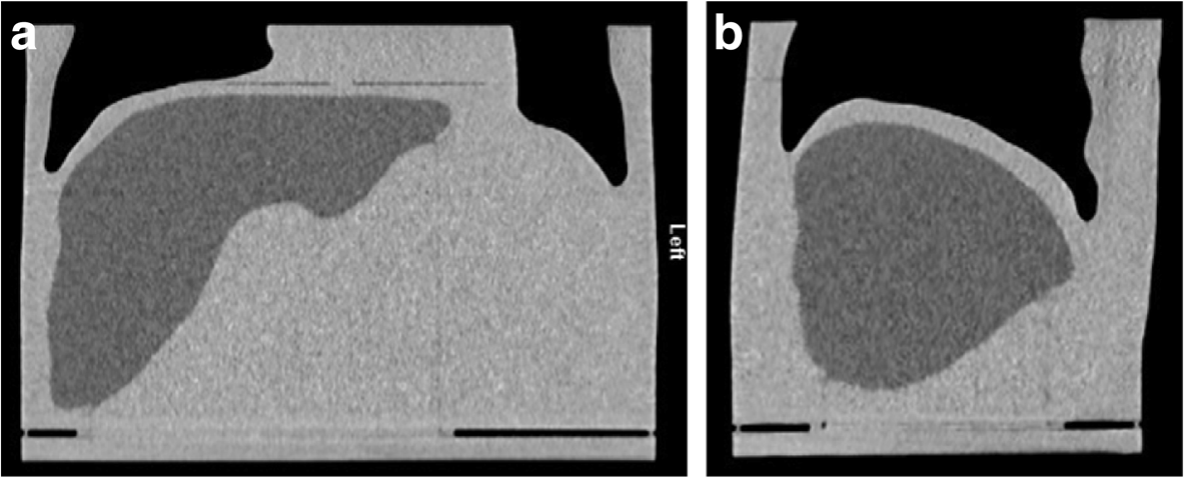
Coronal (**a**) and sagittal (**b**) computed tomography cross sections through the phantom

**Fig. 8:**
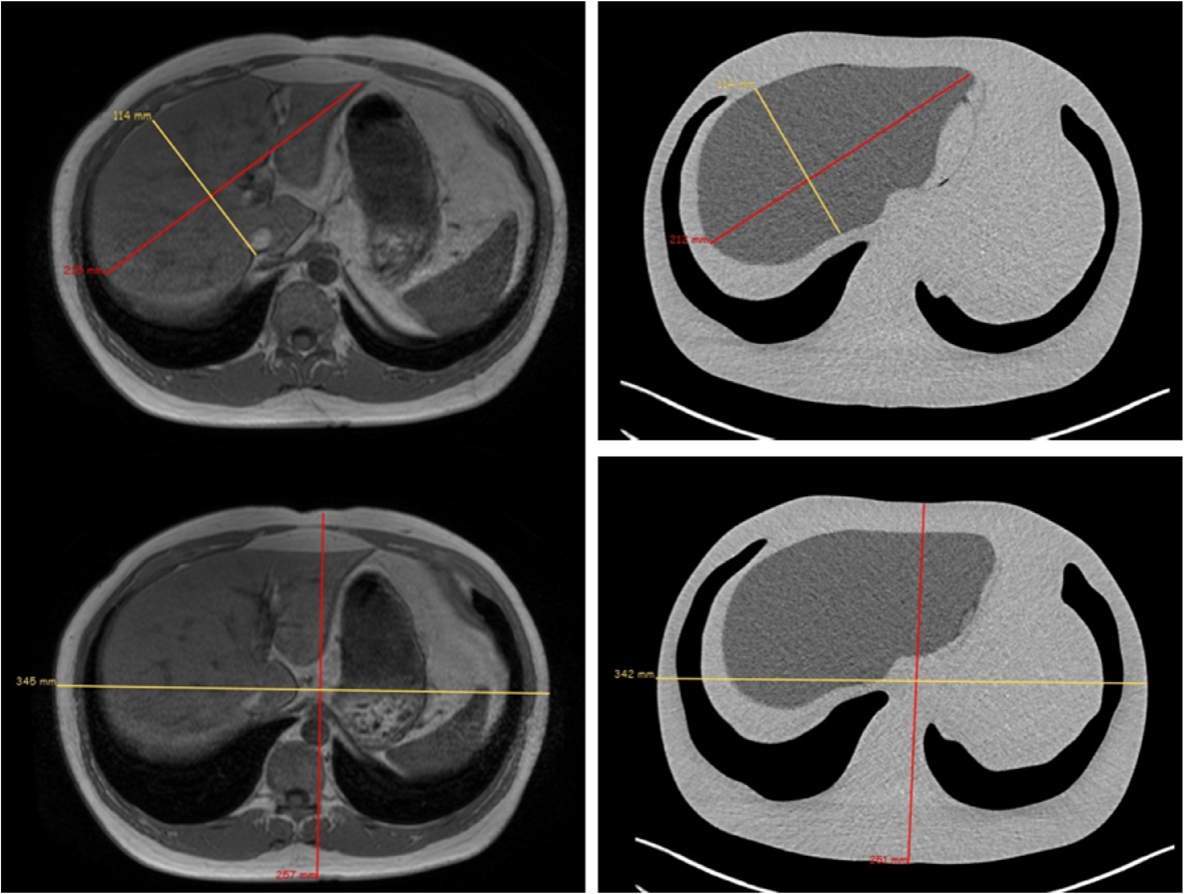
Transaxial computed tomography cross sections through the phantom with measurement lines used for size comparison

Figure 7 shows coronal and sagittal CT slices through the phantom. Transaxial CT images with the corresponding MR slice are given in Fig. 8. Measurement lines along the long and short axes of the liver and abdominal trunk are also given, and the comparison results are summarised in Table 5. The largest factor to affect variation in volume was generated when first smoothing the mesh to remove the image pixilation. The calculated volume of the smoothed liver and the printed phantom was less than 0.5 %. Despite the relative variation in volume between the original MR and phantom, this difference was considered acceptable as the voxelisation and freehand contouring would generate an uncertainty in the original measured organ volume. The final liver volume remained consistent with the cohort average reported by Theysohn et al. [11].

**Table 5:** Phantom dimension measurements compared to the original MRI dimensions

	Original MRI	Phantom	Difference (%)
Liver volume (g)	1972	1783	9.6
Liver long axis (mm)	215	212	1.4
Liver short axis (mm)	114	114	0.0
Trunk anterior/posterior (mm)	257	251	2.3
Trunk left/right (mm)	345	342	0.9

### Phantom imaging and dosimetry

**Fig. 9:**
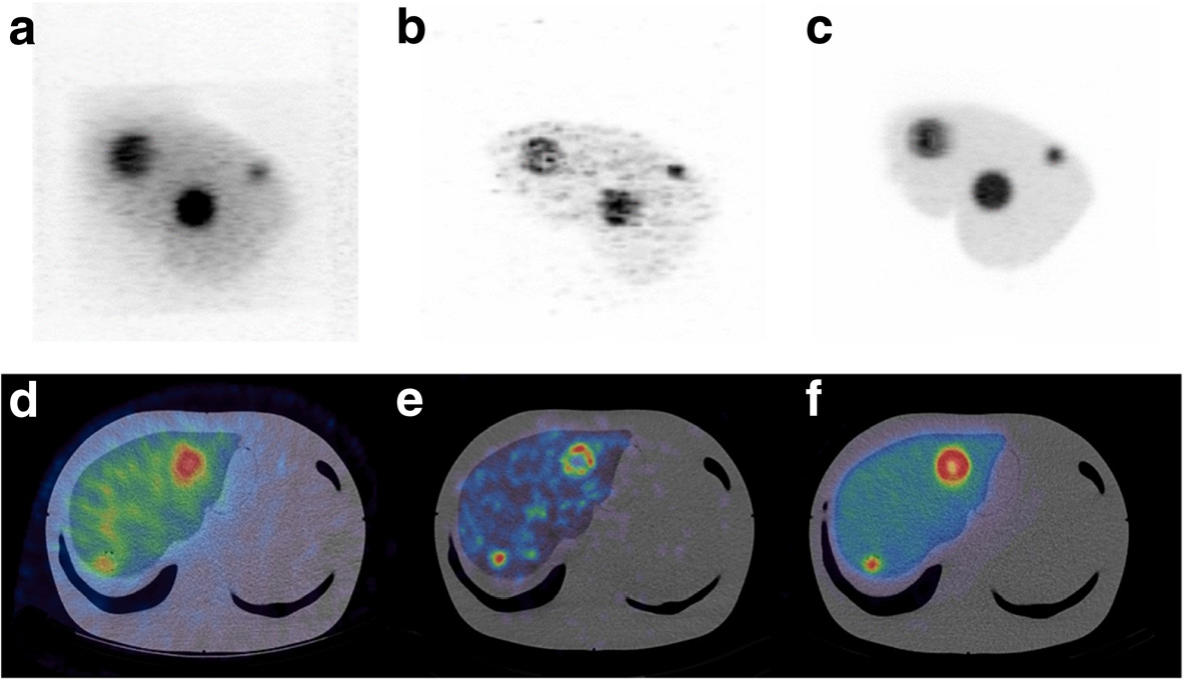
Y-90 SPECT/CT, MIP image (**a**) and fused transaxial slices (**d**). Y-90 PET/CT, MIP image (**b**) and fused transaxial slice (**e**). Tc-99m SPECT/CT, MIP image (**c**) and fused transaxial slice (**f**). Transaxial slices correspond to a plane intersecting the 20- and the 40-mm shell inserts

Figure 9a–c shows maximum intensity projections of the filled phantom imaged with Y-90 SPECT/CT bremsstrahlung, Y-90 PET/CT and Tc-99m SPECT/CT. Figure 9d–f shows the corresponding transaxial SPECT and PET slices through the phantom fused with the CT data. The transaxial slice corresponds to a plane intersecting the 20-mm sphere and the 40-mm shell insert.

Measured lesions and liver activities calculated using the partition model are given in Table 6 for all three imaging modalities with comparisons to the true activity measured during preparation. It can be seen that SPECT overestimates normal liver activity and underestimates lesion activity. Of the three imaging techniques, PET is the most accurate.

**Table 6:** Lesion and liver activities measured within the phantom calculated using the partition model and compared to the true activity measured at preparation

	Activity (MBq)			
	Bremsstrahlung	PET	Tc-99m SPECT	True
40-mm lesion	30.8	51.3	50.7	56.9
40-mm shell lesion	21.3	37.2	33.7	41.2
20-mm lesion	2.6	7.0	5.8	6.9
Liver	590.4	415.6	555.2	500.0

**Fig. 10:**
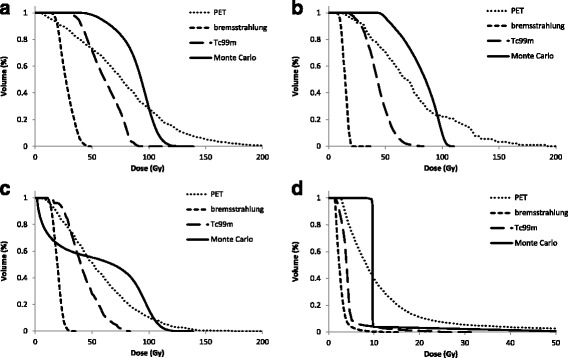
Cumulative dose-volume histograms generated using the local deposition method for the 40-mm lesion (**a**), the 20-mm lesion (**b**), the shell lesion (**c**) and the whole liver (**d**). Comparisons made with those derived from Monte Carlo simulations

Cumulative dose-volume histograms (cDVH) for the three lesions and entire liver volume generated using local deposition for each quantified scintigraphy image are given in Fig. 10 with comparisons to those derived using MC and the known activity within each compartment. In each example, it can be seen that both Tc-99m and bremsstrahlung imaging underestimate the absorbed dose. This underestimation can be contributed to errors in quantification that originate from delineation of the liver volume. For PET imaging, the 50 % cumulative dose-volume is a better match for the MC-derived absorbed dose, indicating superior quantification. However, the shape of the cDVH is very different, and in this respect, the Tc-99m and bremsstrahlung is a better match. All three imaging modalities overestimate the absorbed dose delivered to the centre of the spherical shell lesion (Fig. 10c).

## Discussion

3D printing is an emerging field which has recently gained considerable media attention. A number of medical applications of 3D printing have been proposed including printing of orthodontic appliances [19], prosthetic designs [20] and surgical guides to teach, rehearse and choose treatment strategies [21]. Production of imaging test objects using rapid prototyping has also been suggested. Harrison et al. [22] used a computer numerical control (CNC) milling technique to produce a negative mould used to cast a mixed density anthropomorphic radiotherapy phantom. However, the cost of this approach is prohibitive and does not offer the flexibility of design alterations offered by 3D printing. The main cost of the Abdo-Man phantom was due to the printing of a solid abdominal trunk. This design offered additional strength and ease of filling compared to a shell design, which would have significantly reduced material cost.

Hunt et al. [23] produced a QC phantom designed around the “porous phantom” by DiFilippo et al. [24]. The 3D-printed phantom consisted of a cylindrical matrix of columns of decreasing width and separations. When filled with radioactive solution, changes in the sub resolution columns produced a phantom with sphere inserts of differing size and contrast. However, the porous phantom is limited to fixed geometry and prone to air bubbles and blockages. Holmes et al. [25] created a sub resolution sandwich phantom by placing paper printed with radioactive ink between blocks of 3D-printed material. An anatomical brain phantom was created whereby the source distribution could be altered by changing the printed distribution on the paper. This methodology allows for qualitative image assessment. However, exact source concentration is difficult to calculate, and the printing and assembly of the phantom is time consuming.

In this work, a multi-compartmental anthropomorphic test phantom was developed, based on real anatomy and specific to the patient cohort of interest. The total print time for the phantom was approximately 8 days. Physical properties of the print material fulfilled the initial criteria and were comparable to other commonly used materials. The final structure was watertight, rigid and sufficiently durable to withstand multiple assembly, transport and scanning protocols. To date, the phantom has undergone approximately 20 different acquisition protocols across different institution sites and used by multiple operators. The design of the phantom fulfilled the required brief in that the anatomical detail was representative of the patient cohort and did not significantly differ in size or shape from the original patient. The liver design offered more flexibility for insert placement than commercially available designs yet allowed reproducible construction on reassembly, as demonstrated in Fig. 9.

A potential application of the phantom has been demonstrated in a dosimetry study. Y-90 PET, SPECT and Tc-99m SPECT images of the phantom were obtained and used to estimate absorbed doses to lesions and normal liver. The accuracy of the quantification was determined by comparing the measured activity with the known activity within the phantom measured at preparation. The corresponding absorbed dose map derived from the quantified images was compared against a true absorbed dose map generated using the known activity and MC simulations. Like most commercial dosimetry software, image quantification was achieved using the partition model which is dependent on the operator’s ability to outline the liver volume. The complex anatomical shape of the Abdo-Man liver therefore allowed for a more accurate representation in outlining and hence quantification accuracy compared to that using a geometric alternative. The dosimetry results presented are designed to demonstrate the application of the phantom, and further investigation would be required to validate these initial findings and potentially optimise the methodologies for improved quantification and absorbed dose accuracy. In future work, the phantom will be used to analyse a number of commercial SIRT dosimetry software packages and investigate possible improvements in quantification with alternative reconstruction algorithms, such as the Siemens X-SPECT and Hermes SUV-SPECT software.

The phantom is the first in a range of “Abdo-Man” phantoms to be developed for this application, and unlike conventional manufacturing techniques (which require expensive tooling for mass production), design alterations can easily be implemented before the next phantom is printed. In the future, design evolutions of the phantom are planned; these will include simulation of lung shunts and the addition of lobular cavities within the liver section. Further work is also being undertaken to incorporate bone-mimicking materials. Other applications where this technology could be employed include manufacturing phantoms for use in preclinical scanners, replacement parts for old phantoms and dosimetry phantoms for external beam radiotherapy (in combination with polymer gel technology) [26]. 3D printing offers additional flexibility in design and reduced costs compared to conventional manufacturing techniques. Wider and more routine applications of such phantoms will allow for treatment validation and optimisation and lead to improved outcome for patients.

## Conclusions

An anthropomorphic test phantom based on real patient anatomy and specific to the patient cohort of interest has been manufactured using a 3D printer. The final phantom meets the initial design criteria, and the production material is comparable to standard materials. Production time and cost is significantly reduced compared to standard methods, and designs can offer more flexibility than those previously available. This technology is suitable for a number of applications, and its future use for phantom manufacture could become routine.
